# Recurrence monitoring for ovarian cancer using a cell phone-integrated paper device to measure the ovarian cancer biomarker HE4/CRE ratio in urine

**DOI:** 10.1038/s41598-021-01544-4

**Published:** 2021-11-09

**Authors:** Emily C. Kight, Iftak Hussain, Audrey K. Bowden, Frederick R. Haselton

**Affiliations:** 1 Department of Biomedical Engineering, Vanderbilt University, Nashville, TN 37232 USA; 2 Vanderbilt Biophotonics Center, Vanderbilt University, Nashville, TN 37232 USA; 3 Department of Chemistry, Vanderbilt University, Nashville, TN 37232 USA

**Keywords:** Cancer screening, Urological manifestations, Assay systems, Diagnosis

## Abstract

Ovarian cancer has a poor cure rate and rates of relapse are high. Current recurrence detection is limited by non-specific methods such as blood testing and ultrasound. Based on reports that human epididymis four (HE4) / creatinine (CRE) ratios found in urine are elevated in ovarian cancers, we have developed a paper-based device that combines lateral flow technology and cell phone analysis to quantitatively measure HE4/CRE. Surrogate samples were used to test the performance over clinically expected HE4/CRE ratios. For HE4/CRE ratios of 2 to 47, the percent error was found to be 16.0% on average whether measured by a flatbed scanner or cell phone. There was not a significant difference between the results from the cell phone or scanner. Based on published studies, error in this method was less than the difference required to detect recurrence. This promising new tool, with further development, could be used at home or in low-resource settings to provide timely detection of ovarian cancer recurrence.

## Introduction

Despite treatment, up to 70% of patients with ovarian cancer (OC) will develop recurrence of disease^1^, and patients faced with a high relapse rate are concerned about recurrence. Even though OC patients undergo long-term surveillance to improve recurrence detection, current diagnostic methods, such as pelvic examination, transvaginal ultrasonography, and blood tests such as cancer antigen 125 (CA-125), are invasive and require a clinic visit. Factors such as making an appointment to have blood work done, traveling to the location, a lack of confidence in their symptoms (cramping, bloating, frequent urination), being too busy, not wanting to bother a doctor and the lack of frequency of testing were highlighted concerns of OC patients^2,3^. Currently, there is no at-home monitoring test for ovarian cancer recurrence that can address many of these factors. As many cancer patients eventually relapse, there is a great need to develop at-home detection methods to monitor molecular biomarker levels indicative of recurrence and reduce mortality^4^.

Human epididymis protein 4 (HE4) is a glycoprotein that is highly expressed by ovarian carcinomas^5^. HE4 in serum is overexpressed in ovarian carcinomas and is detected with high sensitivity and specificity^6^. Recent studies have shown that HE4 in serum offers more forecasting power for OC recurrence than CA-125, a biomarker of many cancers^7^. In 2009, FDA approved using serum HE4 levels to monitor ovarian cancer recurrence (OCR) risk, but this method also requires travel to clinic, phlebotomy, and it is expensive^8,9^. Due to the ease of collection of urine, HE4 in urine has emerged as a promising target in ovarian cancer recurrence monitoring at-home. Urine HE4 has been found to be a biomarker for ovarian neoplasms with improved sensitivity in early disease compared to HE4 in serum^10^; additionally, HE4 is detectable in urine earlier than in serum^11^.

Unlike serum HE4, urine HE4 fluctuates with volume, and creatinine (CRE) is commonly used as an internal standard to normalize the ratio of urinary biomarkers^12^ and HE4 in particular^13,14^. The ratio of HE4/CRE has been found to be a better predictor of early and late stage ovarian cancer than urine HE4 alone with highly predictive cut-off range of 3.5^11^. Hellstrom et al. reported higher concentrations of HE4/CRE ratios in ovarian cancer patient urine ranging from − 0.5 to 2.5 for log10 values of HE4/CRE^10^. HE4/CRE can range from 2 in healthy patient samples to nearly 45 (although most are less) in late stage ovarian cancer^10^. Importantly, Liao et al. reported that urine HE4 may also be more useful than serum HE4 in differentiating low malignant potential cysts from early ovarian cancer^15^.

Due to their ease of use, low cost, and robust manufacturing, paper-based tests, such as lateral flow assays (LFAs), are attractive tests for both at-home testing and low-resource testing. Lateral flow assays rely on a colorimetric reaction that can be read by the human eye as a simple yes/no result^16,17^. In these yes/no applications, improving the limits of detection is the major challenge. However, both HE4 and creatinine are present in large quantities and are easily detectable in urine, so a lower threshold is not a concern. In addition to qualitative uses, LFAs have also been used for quantitative detection of various proteins or nucleic acids^18^. The challenge in a paper-based test for HE4/CRE is accurate quantification of HE4 and creatinine values in the clinically relevant ranges. For quantification of LFAs, an optical instrument is required for capturing and measurement of the output. Owing to their portability, low-cost, and widespread use, cell phones are well-studied instruments to quantitate LFAs for home-testing and low-resource setting testing^19–21^.

In this report, we describe the development of an at-home paper-based test to measure both HE4 and creatinine in one device. Based on previous reports, we used surrogate samples containing HE4/CRE tested from 2 to 47 which are within the range of human samples reported by Hellstrom, with particular interest in the initial rising of values of HE4/CRE observed in recurrence. With further validation, this would require only the use of a single diluted urine sample to both test strips for analysis at the same time. The approach is based on standard LFA technology and incorporates a creatinine measurement in a low-cost assay format that can be imaged and quantified using a flatbed scanner as well as a cell phone app.

## Results

**Figure 1:**
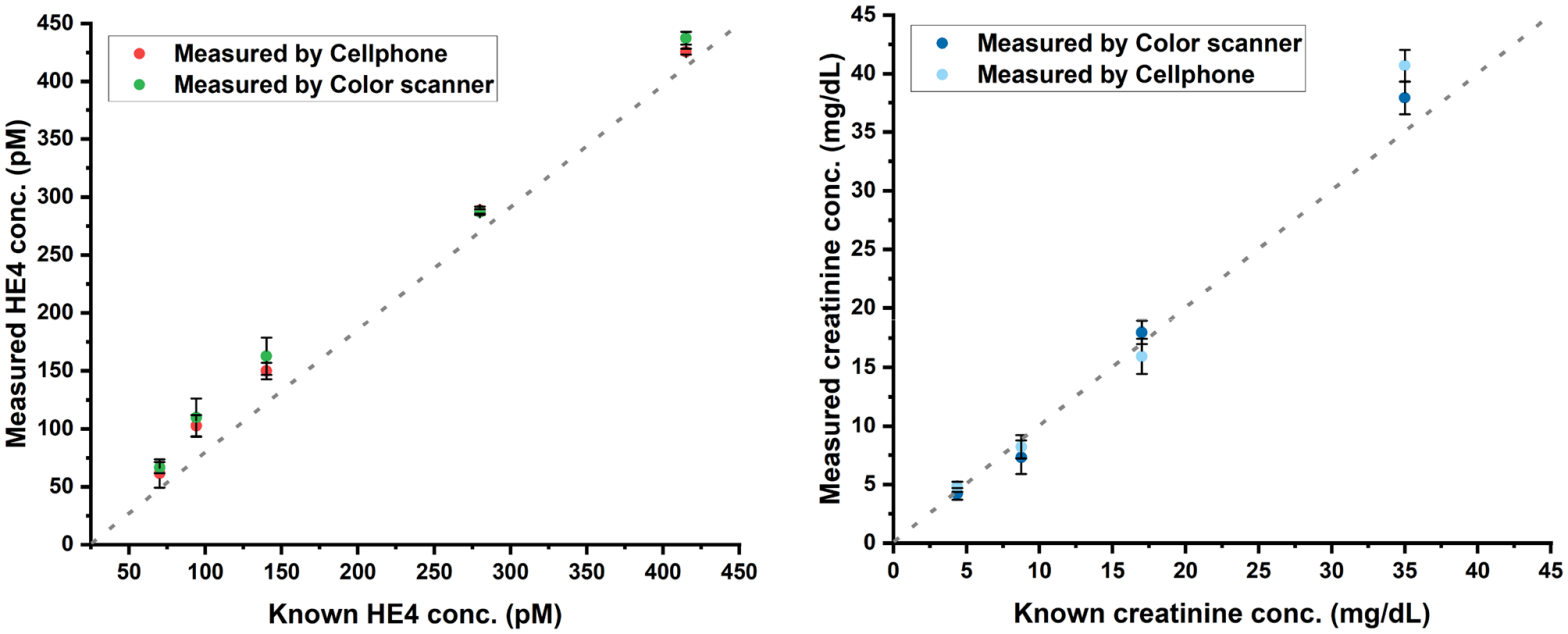
Comparison between flatbed scanner and cell phone measurement of HE4 concentrations (left) and of creatinine concentrations (right). Error bars the percent error found for each concentration (mean + s.d, n = 4). The dotted line indicates the line of identity. Error bars the percent error.

In tests of the accuracy of this approach, we varied the concentration of HE4 or CRE individually (Table 1 in supplemental information). Compared to the known sample concentration, our percent error was usually under 16% for both the scanner and cell phone. Scanner vs cell phone performance is compared to the known values of analyte in Fig. 1. To determine the accuracy for one analyte individually, we varied only the HE4 parameter or only the creatinine parameter along the standard curve for each biomarker. A one-way ANOVA was done to compare the actual value, the scanner and the cell phone for both HE4 and CRE values. For HE4, our calculated F value (0.0095) is smaller than our F critical value (3.8853) (P-value of 0.971). Similarly, for CRE, our calculated F value (0.0042) is smaller than our F critical value (4.2565) (P-value of 0.996). Therefore, the scanner, cell phone and known values are not significantly different for either HE4 or creatinine.

**Figure 2:**
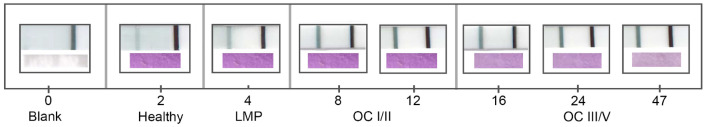
Simulated clinical patient ratios of urinary HE4/CRE. Each image shows a lateral flow strip and corresponding creatinine strip below. In the LFA strip, the left line is the test line and the right line is the control line. *LMP low malignancy potential cyst. The labels below are the ratio of HE4/CRE and the corresponding stage of ovarian cancer given by Liao et al. (2015) and Hellstrom et al. (2010).

**Figure 3:**
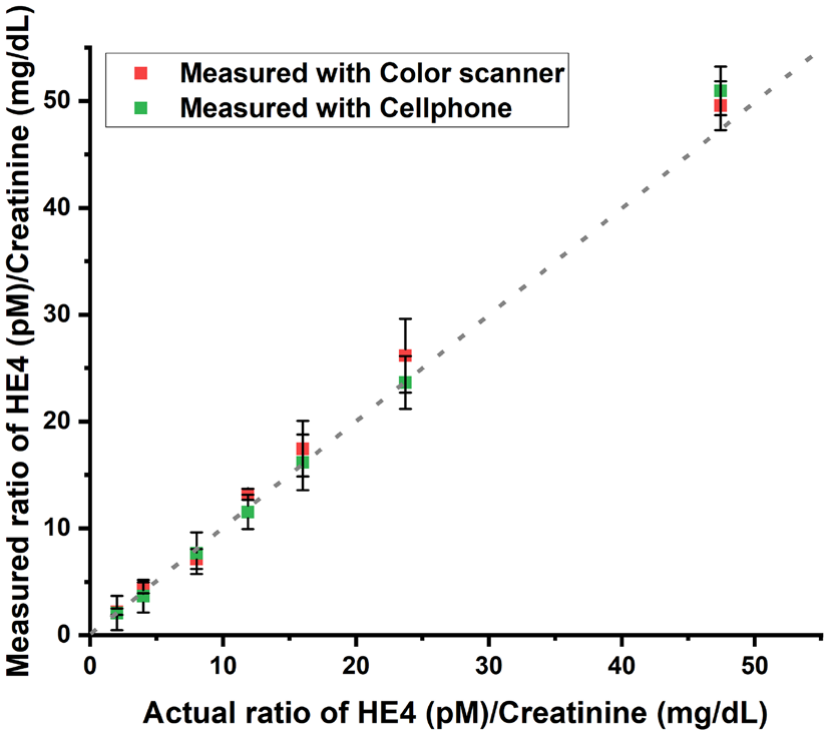
Comparison of mean values for HE4/CRE ratios for scanner and cellphone to actual values (mean + s.d, *n* = *5*). The dotted line indicates the line of identity.

Figure 2 shows flatbed scanner images covering the range of ratios from 2 to 47. Example cell phone images are contained in supplemental Fig. S3. As this figure shows one can qualitatively see the ratio has changed by examining the HE4 test line and creatinine test strip color intensity. However, the images show you cannot get a quantitative answer by visual inspection. The quantitative analysis of images (Fig. 2) are compared to the actual ratio of HE4/CRE and the measured ratios by the scanner and cell phone (Fig. 3). We performed a one-way ANOVA test on the matrix values. Our F value (0.0148) is smaller than our F critical value (3.4028) so there is no significant difference among the actual value, the scanner, and the cell phone (P-value of 0.985). The actual values for the known ratio, the scanner ratio and the cell phone with the associated errors are shown in supplemental Table 2. Our analysis showed that varying creatinine did not significantly alter the intensity of the HE4. Likewise, the level of HE4 did not interfere with the output of the creatinine result.

## Discussion

In this report, we found the method to measure HE4/CRE with a cell phone or scanner worked well over the clinical ranges expected for ovarian cancer recurrence^11^. The intended application for this device is aimed at repeated measurements within the same individual. Based on work by Liao et al., this ratio changes with stage of ovarian cancer. Our expectation is that when applied to a single individual, these ratios would mirror the expected increases found in group average values as reported by Liao. We found the percentage error for the surrogate testing was usually less than 27% for the scanner, with the average percent error being 10%. The worst percent error for the cell phone was better at 12% and was only 4.13% percent error on average for the cell phone. This assumption should be validated with clinical samples. Recurrence was defined by a doubling of the HE4 value in serum^7^. Since urine HE4/CRE correlates positively with serum HE4^10,11,15^, our method has much less error than the expected doubling in recurrence. Therefore, if a user sees a doubling in their HE4/CRE ratio, this is well outside the error range we observed and should be interpreted as a change in their risk of recurrence. These positive preliminarily results lay the groundwork for future longitudinal testing with real clinical samples.

Our results indicate that the cell phone app provides similar results to the flatbed scanner (Figs. 2, 3). The particular brand of flatbed scanner was an ideal comparison to our cell phone app since both devices use LED illumination. Additionally, ImageJ was used to measure the intensity of the LFA and creatinine test. The custom phone app automatically analyzes the intensity of the test, which likely reduces human error compared with the manual measurement method in ImageJ. The cell phone app also produces results much faster compared to the scanner. The custom cell phone app allows a more streamlined and robust process for users as well as reduced cost and space over that required for a flatbed scanner without a significant difference in results. Additionally, the cell phone app analyzed two tests in a single image. This allows us to compare HE4 and CRE, but it could be done for any analyte in a sample. This streamlined process, affordability, and comparable accuracy to the scanner suggests our device shows promise as a valuable platform for home testing.

We chose our HE4/CRE test ranges based on published reports on the levels found in urine in early recurrence. Our values of HE4 concentration are inside the clinical urine ratio ranges published by Hellstrom et al. Since the HE4 concentrations in urine are much higher than the LoD of our assay, our method could be used with higher dilution factors. The normal creatinine range is around 20–275 mg/dL in women. We tested ranges to 4.4 to 35 mg/dL to be able to include the lower concentration ranges of urine based on the need to dilute the urine sample. Hellstrom et al. reported HE4 concentrations in urine of roughly 10–10,000 pM. We tested ranges of 28–543 pM based on the assumption of a 1:40 dilution. Since the intended use of the technology is for recurrence monitoring, where levels will be lower, we focused our testing on the initial rise in ratio.

In contrast with other work by Wang et al.^22^, our device does not require the complicated infrastructure necessary to perform ELISA, making it more adoptable for home-testing. Wang et al. coupled a microchip ELISA with a cell phone mobile application that could detect the HE4 biomarker in urine from ovarian cancer patients. However, the authors did not normalize HE4 to creatinine. Also, an ELISA microplate and ELISA microchip both require numerous sample processing steps (i.e., three washing steps, several hour incubation steps, and manual mixing of the final solution), which increase cost and error. Limited lab infrastructure and skilled technicians are not widely available in low resource settings, which make ELISA and ELISA-based devices difficult to access. Our urine LFA is easily integrated with any cell phone with our customized app. The signal from the test line can be quantified by the custom designed urine LFA, making it possible to detect changes in HE4 level in urine by untrained users. Without the phone, our entire device is under $5.00 to create.

One application for this device is to facilitate biomarker tracking over time with patient-specific baseline HE4/CRE levels. Although many academic papers have demonstrated various devices or methods that can differentiate healthy and OC urine^22^, these devices have not been translatable in a clinical setting^23^. Due to variation of OC (stage, grade or subtype), a single biomarker cut-off value may not very useful at the individual patient level^24^. However, measuring the amount of HE4 urine when first diagnosed with OC and again after debulking surgery and treatment may provide physicians with two critical biomarker points to compare (disease level and no evidence of disease level). By serially monitoring HE4/CRE in urine and comparing patient-specific levels, users may be presented with new biomarker changes to guide further invasive testing.

This type of testing method would be useful in less-developed countries, which face a rapid rise in cancer incidence without increased infrastructure investments in healthcare^25^. Due to this constraint, methods that can detect biomarkers in human samples remotely without the need for training or expensive equipment will have greater adoptability. Low-cost and easy-to-use cancer management strategies would benefit less-developed regions as well as more developed countries. In 2013, the World Health Organization (WHO) endorsed self-sampling as an option for the initial screening for Human Papilloma virus (HPV) for low- to middle-income countries in an effort to reduce cervical cancer death^26^. Studies by Kamath et al. showed that self-sampling for cervical cancer screening in low-resource environments was not just convenient, it was also able to reduce disparities in access to screening and reduce mortality^27^.

Another application of this technology is to monitor early therapy responses. Serial measurements of HE4 in urine show that mean urine HE4 levels remained stable (7% decrease) in patients that proved to be platinum resistant, while they decreased 68% in those that proved to be platinum sensitive^11^. This indicates that urine HE4 levels may also be a useful tool for monitoring recurrence in patient with platinum resistance. Urine HE4 should be studied further to evaluate the predictability of chemotherapy response. One way to do this is by using the proposed method in this report. This remote testing method may indicate the need to switch to a more effective therapy in a timely manner when survival outcomes are higher.

Perhaps the most impactful use of this technology would be in early detection of ovarian cancer. Currently, there is no recommended screening test for ovarian cancer^28^. Since ovarian cancer presents subtly and physicians lack sensitive diagnostic tools, most diagnoses occur in later stages in which treatment options are often limited as well as costly. High-risk individuals (patients with a first-degree relative with ovarian cancer or carrier of a BRCA mutation), often undergo pelvic exams combined with Transvaginal Ultrasound (TVU) and blood test for changes in CA-125 levels even though these strategies have shown no proven benefit for reducing ovarian cancer mortality^29^. Interestingly, one study examined a “2-out-of-3” decision rule that required 2 of 3 serum biomarker tests to be positive. Application of this rule identified 100% of the high-risk cases and showed a false-positive rate of 1.5%^30^. This suggests that multiple positive tests may help identify high risk patients for more follow-up testing at clinics. Longitudinal measurements of HE4 within a single patient with a genetic risk may be useful as an early detection tool for gynecological oncologist. In this use, if women who are at genetic risk would record their HE4 levels 3–4 times a year, a significant rise in HE4 could be used to suggest follow-up screening. Results may help guide decisions on the timing of more invasive procedures such as tissue biopsy or prophylactic ovary removal.

The proposed technology is a good match for lateral flow testing and the high abundance of analytes of interest in the urine. As both HE4 and creatinine are present in high amounts and much higher than the limit of detections in lateral flow tests and we expect the dilutions of urine may be necessary prior to application to test strip. A dilution step presents an additional step for a patient and is more error prone as a result. To offset this, a specific-sized pipette coupled with a pre-aliquoted deionized water vial can be combined easily to help offset errors in measurement. In fact, many pregnancy tests require users to use the included small pipette and deliver only 3–4 drops onto the sample pad. As many women are well accustomed to using such tests, this suggests adding a pre-aliquoted water vial to mix the pipetted urine into would not be an unreasonable step. A step-by-step guide with pictures or a “how to” video on the cell phone app would likely assist users in proper test procedure.

Most commercial LFAs include a spray-dried conjugate release pad in their test format. In our prototype, we did not use an air jet dispenser to spray our reporter onto a release pad. Instead, we combined our sample with the reporter in a single Eppendorf tube for 10 min and spotted onto the sample application pad. Future work may verify if the reporter performs in a spray-dried release pad. Additionally, the temperature needed for creatinine incubation presents another critical limitation in this method. Although making a small compartment with a heating element is feasible, we did not perform this additional step to make it suitable for home use. Without the two 4-min incubation steps, the user must wait an additional 16 min for their result. Additionally, although the LED light in the cell phone cassette was affordable ($0.20 per light), the battery dies quickly if left on for more than 20 min. Automating the light function with the camera function would help alleviate this issue. Therefore, an app that can inform the user of decreasing illumination from a dying LED battery would be a worthwhile effort.

## Methods

### Ethics statement

No human or animal materials were used in this study.

### Reagents

Monoclonal mouse anti-human epididymis protein 4 (HE4) was purchased from HyTest (Turku, Finland). The reporter reagent used in the LFA was BioReady150 nm gold nanoshells by nanoComposix Inc (San Diego, California). Ethyl-N;-(3-dimethylaminopropyl) carbodiimine hydrochloride (EDC) and Sulfo-NHS chemistry was used (sigma Cat# E1769-1G, Thermo, Prod# 24510) to covalently bind the antibody to the nanoshell. Hydroxylamine (Sigma, cat# 467804) was used to quench remaining amine groups during the covalent conjugation protocol.

HE4 antigen standards were provided by Fujirebio Diagnostics Inc. (Malvern, PA). For the enzymatic creatinine reaction, we purchased a Creatinine LiquiColor kit from Stanbio (Boerne, TX). The creatinine test paper used was Whatman no. 3, purchased from Cytiva (Marlborough, MA). The dipping soy wax was purchased from Hearts & Crafts (Brooklyn, NY). Parafilm tape was purchased from Hach (Loveland, CO). Anhydrous creatinine was purchased from Thermo (Prod # C4225, lot# SLCF5841). All solutions were prepared with deionized water unless otherwise noted.

### Lateral flow materials and design

To create the lateral flow assay standards for HE4, we used lyophilized standards in the Fujirebio ELISA kit, which contained HE4 antigen in a phosphate buffered salt solution with bovine serum albumin, an inert yellow dye, and a non-azide antimicrobial preservative. To reconstitute, 1.0 mL of deionized water was added to each standard vial. The vial was vortexed and then allowed to stand at room temperature for 15 min. Prior to use, the vial contents were gently mixed by pipette. Standard dilutions were created from the standards for the simulated patient samples with creatinine solutions or deionized water. Vials were stored at 4° C before and after use as recommended by the manufacturer.

**Figure 4:**
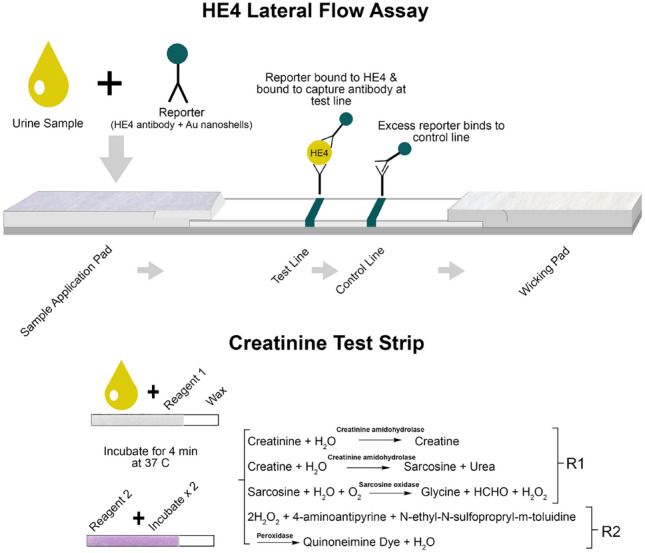
Schematic of the two paper test strips and components. For the HE4 test strip (top), the sandwich assay captures the biomarker in the middle at the test line. The control line is generated when excess reporter binds. For the creatinine test strip, a two reagent system is used to generate a colorimetric reaction with creatinine from light to dark purple. The intensity of the purple color (bottom) indicates the amount of creatinine*.* The two strips are placed in a scanner or cassette for the cell phone to analyze.

Both HE4 and creatinine were measured using paper-based methods (Fig. 4). For HE4, we used standard LFA methods to spot reagents and assemble the test strips. An automatic antibody dispenser (IsoFlow from Imagene Technology Inc) was used to deposit HE4 sheep-derived polyclonal antibody (R&D Systems, Cat# AF6274) across the membrane as the capture antibody at the test line. To make the control line, the antibody dispenser deposited a goat anti-mouse antibody (Lampire, cat# 7455507, Lot# 17H40070) across the membrane. The mouse antibody was chosen for the control line so that the excess mouse antibody would bind to it. The striped membrane was dried for 1 h in an oven at 37° C. Next, the striped membrane was placed onto an adhesive backing card. A sample pad was added to the right edge of the membrane and the wicking pad was added to the left edge, overlapping the membrane by 1 mm to allow for capillary action along the test strip. The assembled card was cut into individual test strips 3.9-mm wide by 70-mm long with an automated guillotine (Matrix 1201 Membrane cutter, Kinematic, Twain hart, CA). The strips were stored at room temperature in sealed pouches with desiccants until use.

Next, we optimized the selection of the reporter antibody and nitrocellulose membranes. To identify the best reporter, we screened three monoclonal HE4 reporter antibodies (R&D systems, LsBio, and Hytest). To test the binding and non-specific binding of each antibody we conducted a dot blot test. We spotted 1 μl spot of polyclonal antibody on the membranes and dried the membrane for 1 h in an oven at 37° C. We then mixed 1 μl of antigen with each of the reporters and spotted the solution onto the sample pad (Supplementary Fig. S1) for the positive control test. For the negative control test, we spotted the reporter without antigen. Non-specific binding occurred when a colored spot was generated without antigen present. Unbound reporters were washed away by the addition of a running buffer to the sample pad. Since the Hytest antibody showed strong signal intensity in antigen binding in the positive control tests and the least non-specific binding in the negative control tests, it was chosen for further testing.

We screened three different nitrocellulose membranes (supplementary Fig. S1) and subsequently chose MDI CNPC (10 μm) membranes (MDI membrane technologies) since they exhibited the least non-specific binding in the dot blot test, and the antigen signal intensity was higher in the positive control tests.

To run the LFA HE4 test, we used 20 μl of nanoshell + HE4 antibody incubated with 25 μl of sample for 10 min on a rotator prior to pipetting it onto the sample pad of the lateral flow test strip. After deposition onto the sample pad, the lateral flow test was left at room temperature for 20 min before measurement on a flatbed scanner or cell phone.

### Creatinine test design

For the design and fabrication of the creatinine test, we used an enzymatic creatinine kit that comprises a two-reagent system that is typically found in automated analysis equipment^31^. Since chemicals can interfere with creatinine determination using the Jaffe reaction^32^, we chose to use enzymatic reagents to measure creatinine. Enzymatic reaction has been chosen over the picrate reaction for the determination of creatinine in clinical labs as the results of enzymatic methods have been reported to match the gold standard method more closely, isotope-dilution-mass spectrometry (IDMS)^31,33,34^. In the first reaction mixture, reagent 1 (R1), the creatinine amidohydrolase, converts creatine to sarcosine, and the oxidation of sarcosine by sarcosine oxidase produces hydrogen peroxide. The hydrogen peroxide generated in R1 is then reacted with reagent 2 (R2) in the presence of peroxidase to react with Quinoneimine dye as shown in Fig. 4. This reaction results in a light to dark purple dye in which the dark purple corresponds to high creatinine concentration in the sample.

Whatman no. 3 paper was used for its high absorbency, medium porosity and medium flow rate. To create the creatinine paper strips, Whatman no. 3 paper was cut with scissors into individual 2.5-mm long by 10-mm wide pieces. The test strip end was then briefly dipped in heated liquid soy wax. The wax end created a barrier for the chemical products generated during the chemical reaction^31^ and allowed for tweezer handling of the test strips.

As powdered creatinine dissolves readily in deionized water, we created a stock solution of creatinine at 80 mg/dL. Standard dilutions were made from the stock solution with deionized water into individual Eppendorf tubes and refrigerated at 4° C until use. The creatinine enzymatic kit uses a 300 μl cuvette to measure absorbance and the manufacturer instructions suggest using 270 μl of R1 and 90 μl of R2. For our small paper-based tests, we reduced the volumes for the two creatinine enzymatic reagents (R1 and R2) but preserved the ratio as suggested by the manufacturer. This reduction in volume was necessary to prevent leaching out of the paper onto the cell phone housing or scanner bed during measurement. We pipetted 18 μl of R1 onto the test strip followed by 6 μl of sample. The test strip was then placed in a 37° C oven for 4 min. Then the test strip was removed from the oven, and 6 μl of R2 was pipetted on the test strip. The test strip was then placed back into the 37° C oven for 4 min. The incubation in the oven was used to decrease the time to measurement: if left at room temperature, the result can be read in 30 minutes^35^. The test strip was finally removed from the oven and allowed to sit at room temperature before measurement on the flatbed scanner and cell phone. To prevent leaching out, a small 3 mm × 11 mm section of Parafilm tape (Hach, Loveland, CO) was added to the phone cassette well where the creatinine test is placed. The hydrophobicity of Parafilm tape ensures that the liquid remains inside the creatinine test strip during measurement.

### Quantification of paper-based tests

The quantitative LFA method involves measuring the amount of an analyte in a sample against a standard curve of known amounts of the analyte^36^. To measure the intensity of the reaction in both the LFA and creatinine tests, we used a CanoScan LIDE 300 scanner from Canon (Ōta, Tokyo, Japan) and a cell phone (Samsung galaxy s20).

In this report, we compare quantification using our cell phone app to a flatbed scanner as the “gold standard”. Flatbed scanners have been used widely to quantitate LFAs and other colorimetric tests^37^. For the scanner measurement of HE4, the individual paper strips were placed face down. The auto scan button was pressed to create a PDF scan. The document was then opened in Adobe Photoshop to convert the PDF to JPEG. The JPEG was then opened in ImageJ. The test line in the image was measured with the "Measure tool”. The output of the measured result was then copied and pasted into Excel for analysis.

**Figure 5:**
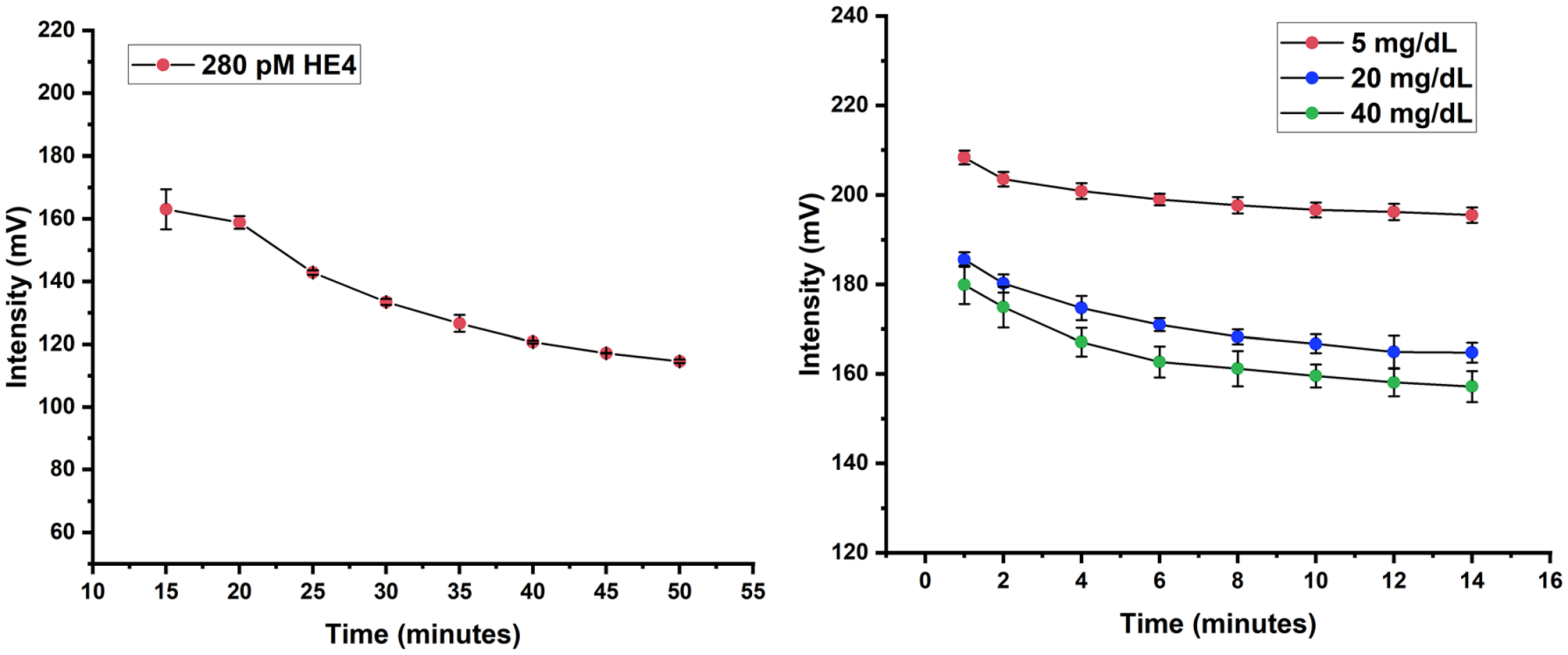
HE4 test line intensity as a function of the assay read time (mean + s.d., n = 2) (left panel). The color intensity as a function of assay read time for three creatinine concentrations of 5 mg/dL, 20 mg/dL, and 40 mg/dL (mean + s.d, n = 3) (right panel).

In this study, determination of HE4 concentration was based on a sandwich assay format in a lateral flow method. Therefore, the timing of when the reaction is considered “complete” and ready to read on a scanner or cell phone app influences the intensity values. Per the manufacturer’s instructions for the membrane of choice, MDI-10, the strip can be read at 15 min after sample application. Therefore, we analyzed the intensity associated with a concentration of 280 pM (the medium standard) over 5-min intervals from the earliest it could be read (Fig. 5). We chose the 20-min time mark because subsequent drying of the test reduced the intensity of the test value by about 12% as shown in Fig. 5.

To determine the optimal time for reading the creatinine test strips, three concentrations of a standard curve were analyzed every 2 min until 14 min. The time point at 8–10 min was selected as the change from 8 to 16 min is less than 2% change (Fig. 5**).**

**Figure 6:**
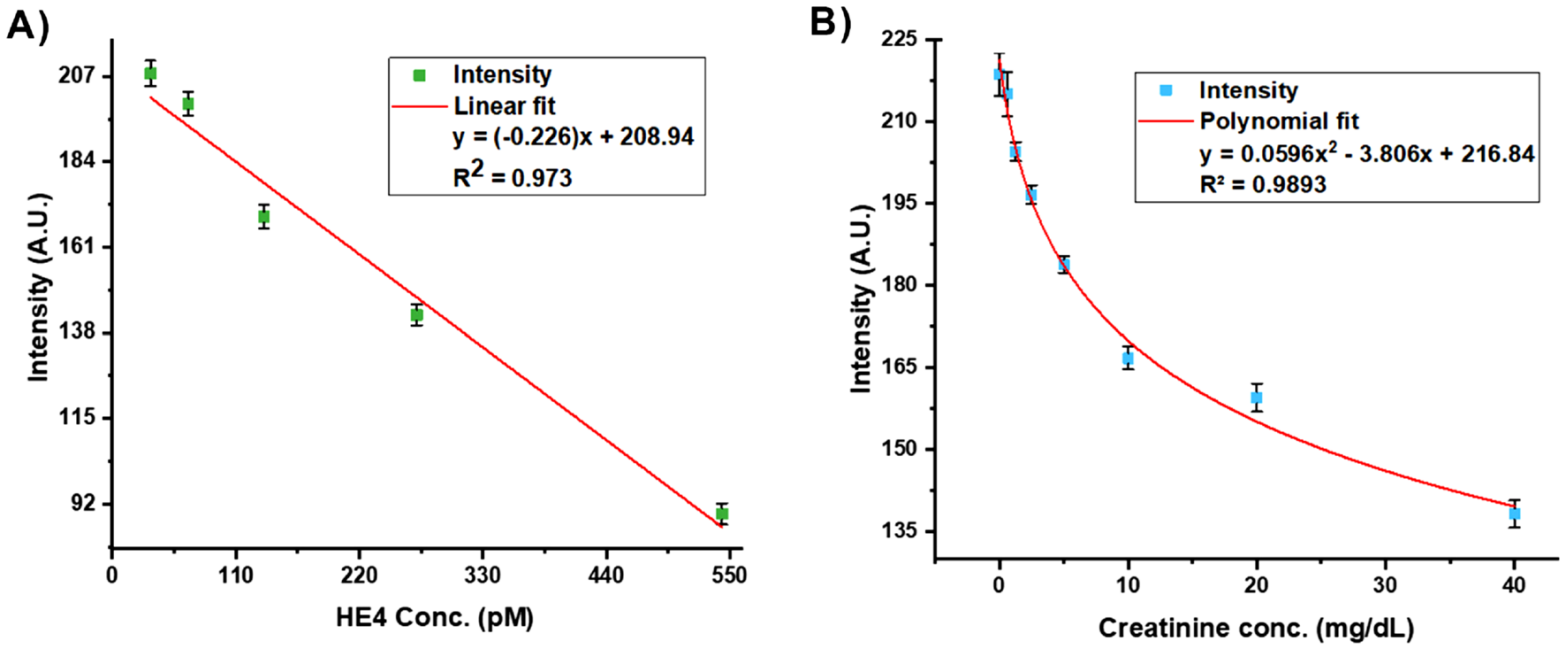
Calibration plots generated from the scanner. (**A**) Standard curve for concentrations of HE4 (pM) (mean + s.d, n = 2). (**B**) Standard curve for concentrations of creatinine (mg/dL) (mean + s.d, n = 3).

To make a standard curve for HE4, we serially diluted the 573 pM HE4 standard from the ELISA kit. We then compared the output of the value calculated from the standard curve to the known value (Fig. 6A). For the scanner measurement of Creatinine, an 80 mg/dL stock solution of creatinine was made and serially diluted to create a standard curve for creatinine. After incubation, the test strips were allowed to sit at room temperature for 8–10 min before being measured in the scanner. Creatinine concentrations up to 40 mg/dL were analyzed, and triplicate assays were performed for each concentration. The results demonstrated a second order polynomial fit range from 0 mg/dL to 40 mg/dL of creatinine (r^2^ = 0.9893), as shown in Fig. 6B. We determined the limit of detection (LOD) of each test strip by analyzing a blank 5 times and calculated a 3 SD limit^38^. The LOD for HE4 is 15 pM and for creatinine test the LOD is 1.2 mg/dL. Average intensity of the blank for is 226.4856 ± 2.9178 for HE4 and 243.31 ± 2.99 for creatinine (Fig. 2).

**Figure 7:**
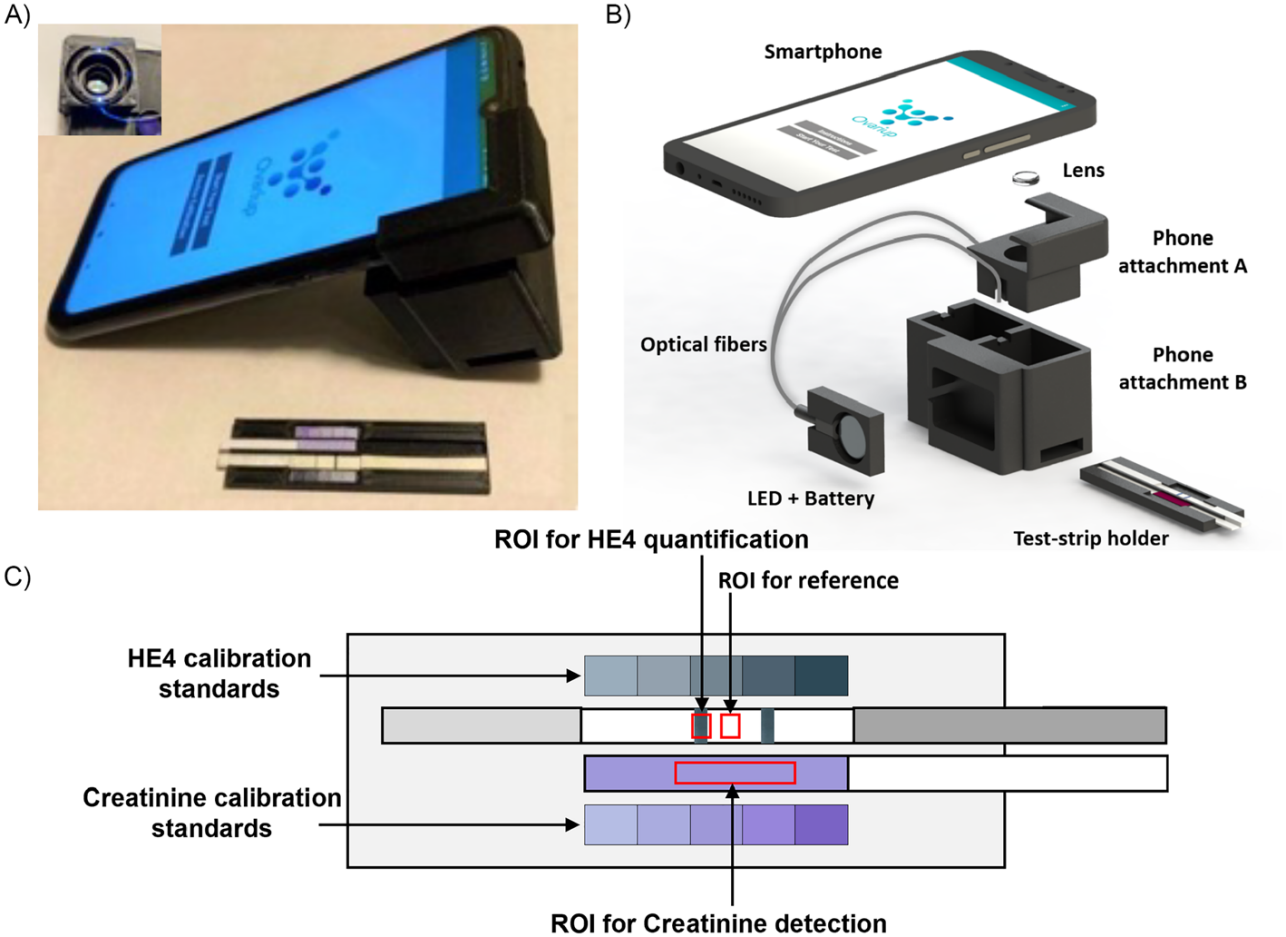
Overview of the smartphone quantification system. (**A**) Image of the prototype components (lens, cell phone attachments and strip cassette). (**B**) A CAD model providing an overview of the components used in developing the system. (**C**) Schematic of the test-strip holder indicating the color detection scheme and the red boxes (arrows) indicate the region of interest (ROI) for HE4, for background reference, and a ROI for CRE.

In addition to quantification with a flatbed scanner, we also developed a cell phone-based system for quantification of both HE4 and creatinine. Figure 7A shows a photograph of the developed device, which includes a 3D-printed black enclosure and a test strip holder. The black enclosure is used to mitigate the problem with varying external lighting conditions. The current system was implemented on an Android platform-based cell phone (Redmi Note 7 from Xiaomi, camera resolution 4000 pixels × 3000 pixels), but the same system can be used with other cell phones with a small adjustment in the design of the phone attachment. As shown in Fig. 7B, an inexpensive plano-convex lens of focal length 75 mm was used to magnify the area of the test strips. The lens was attached to the phone attachment A, which is mechanically connected to the cell phone. Phone attachment B is connected to attachment A. The attachments are positioned perpendicular to the phone to eliminate the need to tilt the phone and to standardize the focal distance to reduce imaging inconsistencies. Both attachments are 3D-printed with black polylactic acid (PLA) material. The material of choice is not significant. Black was chosen to decrease the reflectance of light inside the test strip compartment. Once the test-strip holder is positioned inside of Phone attachment B, ambient light is blocked from entering the inside of the phone attachment B.

It is possible to use a cell phone flashlight as a light source for colorimetric applications^39^. However, in the current application, it is not possible to use the flashlight to illuminate the test strips. This is attributed due to the use of the external lens to acquire an image with a large field of view to cover both the test strips and the calibration standards. Another problem in using the flashlight is that its position varies from phone to phone, therefore the illumination over the detection area will be different for different brands of cell phones^40^. Instead, here we achieve uniform illumination by using a novel optical fiber-based illumination scheme as shown in Fig. S2. Light from a low-cost external battery-powered white LED (Finware LED, Amazon) is coupled to two plastic multimode optical fibers (980-µm core diameter, Edmund Optics), and the other ends of the optical fibers are connected to the phone attachment. The distances between the test strips and the optical fibers were optimized to achieve a uniform illumination area. The battery and the LED compartment are located on the outside of the attachment so that they are easily operable by the user and the battery. While the cell phone battery can be used to power an external LED^41^ but we have observed that long term use of cell phone battery for powering external components heats up the phone and the output power of the LED is significantly reduced when the cell phone battery power is low (< 30%). Therefore, in the current application, we have used an external coin cell battery to power the LED, which can easily be replaced when needed.

Figure 7C shows the schematic of the test-strip holder. The test strips are inserted from the side and manually slid into the field of view. The test-strip holder contains the HE4 test, CRE test and calibration stickers to allow for simultaneously measurement of both tests and calibration stickers. We prepared calibration stickers indicating the color of both the HE4 and CRE standard solutions and attached them to the test-strip holder. The phone application captures both the test strips and the calibration stickers together in a single image frame to evaluates the intensity values. The intensity values from the calibration stickers are used to generate the respective calibration equations and the unknown HE4 and creatinine concentrations are evaluated by interpolating corresponding intensities values in respective calibration equations. Our device evaluates the intensity of both tests and determines the corresponding concentration value by using the calibration sticker as a reference standard. The calibration standards are printed as a vinyl sticker to increase stability over time compared to typical inkjet printer paper. To facilitate on-board data processing, an Android application (app) was developed and installed on the cell phone. The detailed procedures for using this app are given in the supplementary information. To simulate an at-home user, both HE4 and creatinine were measured simultaneously on both the scanner and cell phone imaging modality (Fig. 3).

**Figure 8:**
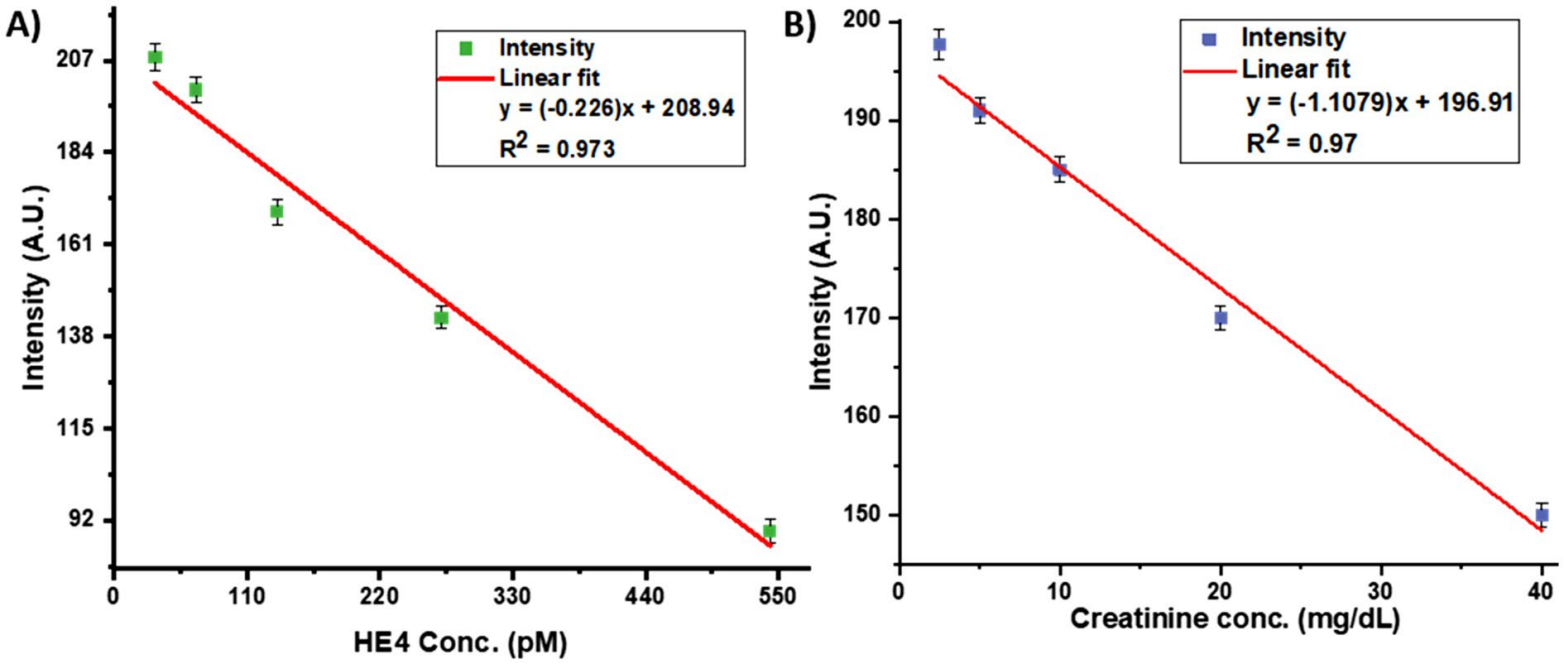
Calibration plots generated from the cell phone-based device. (**A**) Standard curve for concentrations of HE4 (pM) (mean + s.d, n = 2). (**B**) Standard curve for concentrations of creatinine (mg/dL) from cell phone (mean + s.d, n = 3).

Figure 6 shows the calibration plots generated from the HE4 (pM) and CRE (mg/dL) standard solutions using the cell phone app. The analysis algorithm in the phone is simple, as the concentration can be determined by a linear relationship. In the case of HE4 we have selected the concentration range of 33.75 pM-543 pM, which indicates a co-efficient of determination (R^2^) of 0.973 as shown by Fig. 8A. This range was within the range of the ELISA kit. Similarly, for CRE, we evaluated a concentration range of 2.5–40 mg/dL, which shows a co-efficient of determination (R^2^) of 0.97 as shown in Fig. 8B. For the phone, a linear fit was chosen over a polynomial fit since the linear fit provided a high R^2^ value and was computationally easier since there is only one answer given from the intensity calculation.

### Surrogate sample preparation

In this work, we tested the performance of our test using clinically relevant ratios of ovarian cancer recurrence. Surrogate patient samples were created based on previously published work by Liao et al., who measured patient HE4 urine values in pM and creatinine values in mg/dL as a ratio^11^. The range of 2–47 ratios was selected to ensure that the paper-based test was operating in the range of ratio values associated with recurrence testing in urine^11^. Because both creatinine and HE4 can be present in high amounts, Liao et al. diluted clinical samples by 1:40 prior to testing^11^. Therefore, we assume the same level of dilution for our test method and CRE values from 0 to 40 mg/day^31^ were selected for testing as they are in the ranges of diluted urine samples as described by others^10,11^. To create simulated patient samples, we made a 3 × 3 matrix of HE4/CRE solutions. For the three HE4 solutions, we included a high, medium, and low CRE version of each of the HE4 concentrations.

## Conclusion

The availability of a urine-based screening test for ovarian cancer recurrence that is simple enough to perform at home would offer a convenient and less harmful sampling procedure, especially for repeated testing over an extended time. We have demonstrated an improved method to detect changes in urine HE4/CRE accurately at-home. In addition, due to its low-cost, this method can also be employed in low-resource settings where clinical visits are even more difficult. In the future, the methods demonstrated here could be evaluated with patient samples to further validate the clinical performance of the device.

## Supplementary Information

Supplementary Information.
