# Supplementary material for: Recurrence monitoring for ovarian cancer using a cell phone-integrated paper device to measure the ovarian cancer biomarker HE4/CRE ratio in urine
Source: Sci Rep. 2021 Nov 9;11:21945. doi: 10.1038/s41598-021-01544-4 (PMC8578327; doi:10.1038/s41598-021-01544-4)
Supplement: Supplementary file 1 — Supplementary Information. [file 41598_2021_1544_MOESM1_ESM.docx]

**Supplementary Materials**

**Recurrence monitoring for ovarian cancer using cell phone integrated paper device to measure the ovarian cancer biomarker HE4/CRE ratio in urine**

Emily Kight^1^, Iftak Hussain^1,2^, Audrey K. Bowden^1,2^, and Frederick R Haselton^1,3^*

**Supplementary Figures**


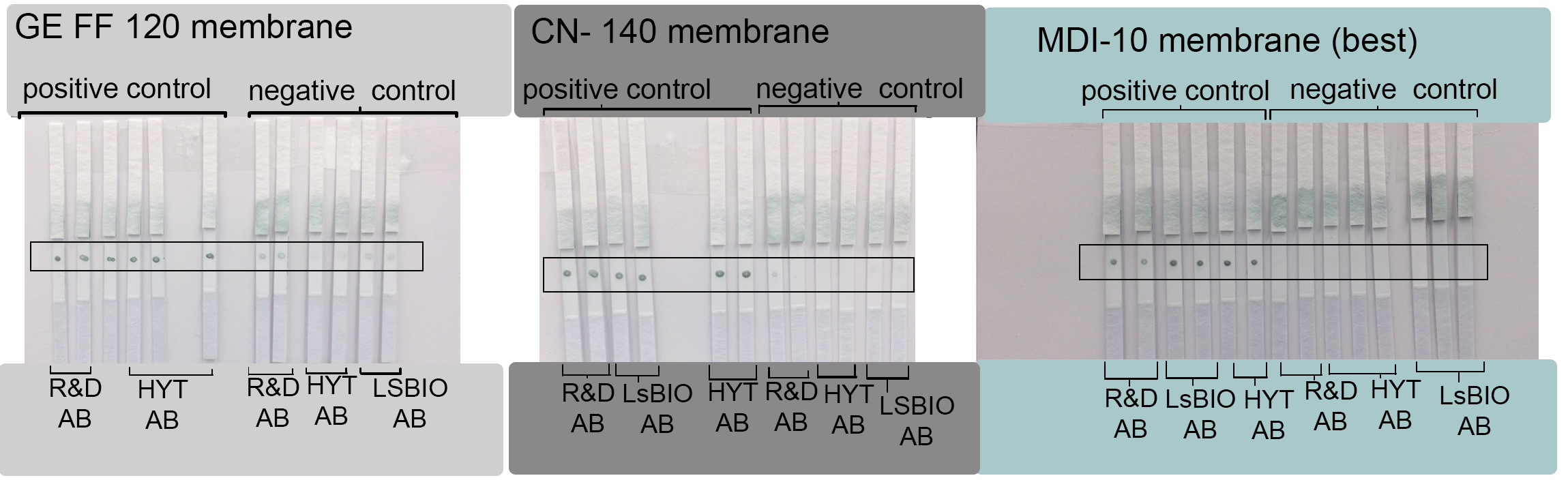


**Figure S1**. Dot blot test was conducted with three different antibody and three different membranes. For the three antibodies tested, MDI-10 showed the least non-specific binding for the control spots and strong signal for the positive spots.


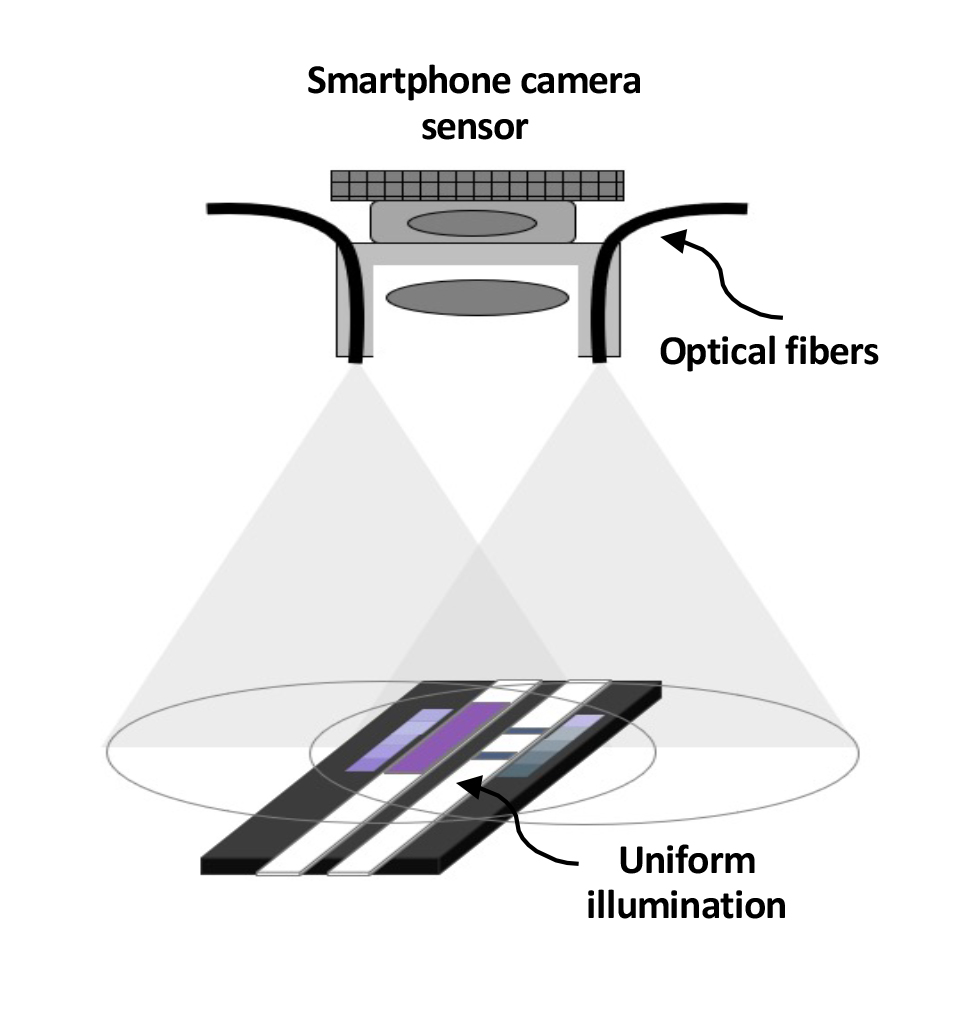


**Figure S2.** Details of the optical fiber-based illumination scheme used to provide uniform illumination over the field of view of the camera.

**Cell Phone app operation.** The cell phone app was developed using MIT app inventor 2: a cloud based open-source platform [38]. This app is compatible with any cell phone with an operating system of greater than 2.3 (Gingerbread). The workflow of the app is shown in **Figure S3**. **Figure S3A** shows the main window of the app, it provides two buttons to the user “Start Your Test” and “Device Calibration”. On clicking the “Start Your Test” button, it will open the test window which contains a live camera view with floating positioning markers as shown in **Figure** **S3B**. The user needs to position the test-strip holder in the device so that the HE4 test line and creatinine test-strip reaction areas are within the boundary of the rectangular markers on the cell phone screen as shown in **Figure S3C**. Now, on clicking the “Capture” button, the app will pop-up the in-built phone camera and capture an image of the test strips as shown in **Figure S3D**. After capturing the image the app will process the image internally and calculate the grayscale intensity of the HE4 test line, the white area of the HE4 test for reference, creatinine test strip and the intensity of the calibrations stickers marked by the circles. As shown in **Figure** **S3E**, the app will change the place holders texts for the intensity values to the grayscale intensity respectively. On clicking the “Analyze Data” button, the application generates the calibration equation from the intensity values of HE4 and creatinine standards as shown in **Figure S3F** and determines the unknown concentration by fitting the intensity values of the biomarkers. Although, the app is self-content to calibration from the calibration stickers but there are situations where a device calibration in needed with freshly prepared standard solutions. To facilitate on-site calibration, the user need to click on the “Device Calibration” button on the home screen, which will open he calibration window as shown in **Figure S3G**. In this window, the user types the standard concentration values for HE4 and the corresponding intensity values into the respective boxes. After inserting the values, the user clicks the “Calibration for HE4” button, the app performs the least square fitting and generates the calibration equation as shown in **Figure S3H**. After that on clicking the “Save” button shown in **Figure S3I**, the calibration curve can be implemented to the app data processing algorithms. The same procedure can be used to calibrate the device with creatine standards. A copy of the code is provided by request.


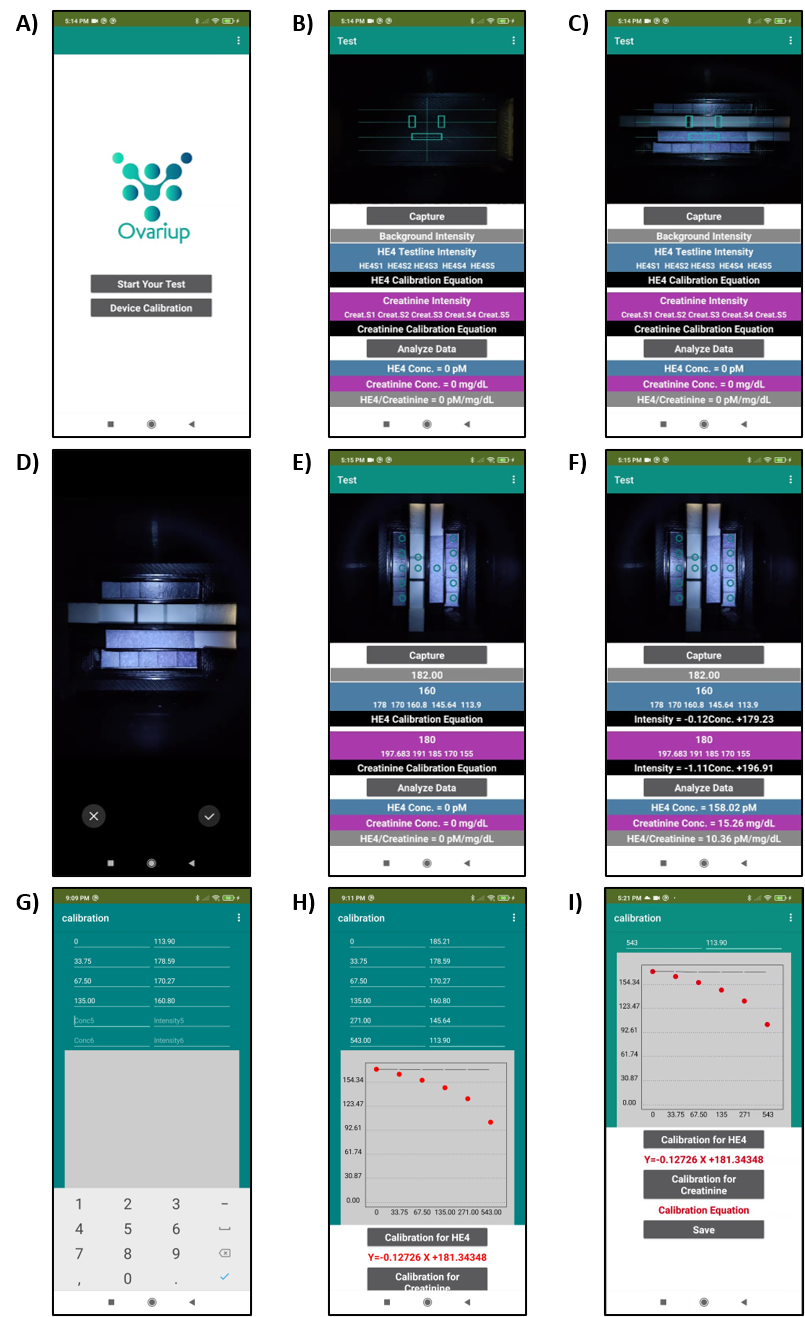


**Figure S3.** Workflow of the cell phone app.

**Supplementary Tables**

| **Known Concentration** | **Scanner Concentration** | **% error** | **Cell phone Concentration** | **% error** |
| --- | --- | --- | --- | --- |
| 4.4 mg/dL CRE | 4.8 mg/dL CRE | 9.9 | 4.2 mg/dL CRE | 4.0 |
| 8.8 mg/dL CRE | 8.2 mg/dL CRE | 6.2 | 7.3 mg/dL CRE | 16.4 |
| 17.5 mg/dL CRE | 15.9 mg/dL CRE | 8.9 | 17.9mg/dL CRE | 5.8 |
| 35.0 mg/dL CRE | 40.7 mg/dL CRE | 14.1 | 37.9mg/dL CRE | 8.3 |
| 70 pM HE4 | 66 pM HE4 | 4.8 | 61 pM HE4 | 12.2 |
| 94 pM HE4 | 109 pM HE4 | 16.6 | 102 pM HE4 | 9.2 |
| 140 pM HE4 | 162 pM HE4 | 16.2 | 149 pM HE4 | 7.1 |
| 280 pM HE4 | 287 pM HE4 | 2.5 | 288 pM HE4 | 3.1 |
| 415 pM HE4 | 437 pM HE4 | 5.4 | 425 pM HE4 | 2.6 |

**Table 1.** CRE (n=3) and HE4 (n=4) concentrations were calculated from the standard curve using the flatbed scanner and cell phone and compared to the actual known value. Percent error for all concentrations is reported.

| **Known Ratio** | **Scanner Ratio** | **% error** | **Cell phone ratio** | **% error** |
| --- | --- | --- | --- | --- |
| 2 | 2.2 | 9.77 | 2.1 | 3.74 |
| 4 | 4.4 | 11.06 | 3.6 | 8.74 |
| 8 | 7.1 | 10.72 | 7.7 | 5.02 |
| 11.9 | 13.2 | 11.33 | 11.5 | 2.67 |
| 16 | 17.4 | 9.04 | 16.2 | 1.08 |
| 23.7 | 23.7 | 10.33 | 23.6 | 0.28 |
| 47.4 | 49.6 | 4.50 | 50.9 | 7.40 |

**Table 2.** Surrogate patient samples created for three HE4 concentrations across three different creatinine concentrations. Scanner and cell phone values are reported for each biomarker. Percent error is reported for each concentration (n =5). Duplicate ratios are not shown.
